# Examining the Link Between Problematic Smartphone Use and Substance Use Disorders Among College Students: Association Patterns Using Network Analysis

**DOI:** 10.3390/ijerph22070973

**Published:** 2025-06-20

**Authors:** Amanda Severo Lins Vitta, Wanderlei Abadio de Oliveira, Lucio Garcia de Oliveira, Laura Soares da Silva, Évelin Moreira Freires, Fernando Ferreira Semolini, Makilim Nunes Baptista, Claudio Romualdo, Hyoun S. Kim, Denise de Micheli, Adriana Scatena, André Luiz Monezi Andrade

**Affiliations:** 1 Graduate Program in Psychology, School of Life Sciences, Pontifical Catholic University of Campinas, John Boyd Dunlop Avenue, Ipaussurama Garden, Campinas 13060-904, SP, Brazil; severolins.amanda@gmail.com (A.S.L.V.); wanderleio@hotmail.com (W.A.d.O.); laursoaresilv@hotmail.com (L.S.d.S.); claudio.romualdo42@gmail.com (C.R.); 2 Department of Neuroscience, School of Medicine, ABC Fundation, 200 Lauro Gomes Avenue, Santo André 09060-870, SP, Brazil; lucgoliver@gmail.com (L.G.d.O.); evelin.mfreires@gmail.com (É.M.F.); 3 Department of Psychology, Ryerson University, 350 Victoria Street, Toronto, ON M5B 2K3, Canada; fernando.semolini1@gmail.com (F.F.S.); andrewhs.kim@torontomu.ca (H.S.K.); 4 Department of Psychobiology, Federal University of Sao Paulo, 1038 Napoleao de Barros Street, Sao Paulo 04023-062, SP, Brazil; makilim.nunes@puc-campinas.edu.br (M.N.B.); demicheli.unifesp@gmail.com (D.d.M.); adriana.scatena7@gmail.com (A.S.)

**Keywords:** smartphone problematic use, substance use disorder, emotional problems, colleges

## Abstract

This study examines the interplay between problematic smartphone use (PSU) and substance use disorders (SUDs) among Brazilian college students, also addressing associated emotional distress (e.g., depression, anxiety, and stress). A total of 3130 students (M_age = 23.6; SD_age = 5.34) participated in an online survey featuring validated measures for assessing PSU, alcohol and drug consumption, and emotional distress. Participants were categorized into problematic smartphone use (PSU) and non-problematic use (nPSU) groups. The prevalence of PSU was 46.9%, notably higher among female students, those lacking a religious affiliation, individuals living alone, and the unemployed. PSU individuals showed significantly elevated rates of alcohol, cannabis, and other illicit substance use, along with greater emotional distress. Network analysis revealed that weekly alcohol consumption and stress symptoms exhibited the highest centrality indices (e.g., stress with high betweenness; alcohol with strong expected influence), underscoring their key roles in connecting PSU and SUDs. These findings suggest that PSU and SUDs may share underlying emotional vulnerabilities, highlighting the need for integrated intervention strategies targeting both conditions concurrently.

## 1. Introduction

Substance use disorder (SUD) is a persistent inability to control the use of psychoactive substances, which can lead to significant issues in physical health, relationships, and performance in work or school [1]. It is well established that SUD is linked to the dysfunctional activation of brain reward circuits, especially in the mesolimbic and mesocortical pathways. This overactivity fuels repeated substance-seeking behaviors [2]. The process disrupts emotional, cognitive, and behavioral regulation, creating a cycle of positive reinforcement—connected to the initial pleasure derived from using substances—and negative reinforcement—related to easing withdrawal symptoms and emotional distress [3].

Recently, some authors have started investigating the connection between substance use disorders (SUDs) and digital addictions, particularly problematic smartphone use (PSU), since this behavior is becoming increasingly common in our digital age [4,5,6]. Problematic smartphone use (PSU) refers to excessive engagement with smartphones that negatively affects daily functioning and mental health, although it may not meet the criteria for formal addiction. Unlike smartphone addiction, PSU encompasses a broader range of problematic behaviors that vary in severity and clinical significance [7]. Previous studies have consistently shown a link between PSU, substance use disorders, and emotional distress. Specifically, some studies have indicated that PSU is associated with increased consumption of alcohol and cannabis [8,9,10]. However, many of these studies focused on a limited range of substances or utilized simplified measures of substance use, which constrains our understanding of how these behaviors interact.

Network analysis has proven to be an effective method for exploring comorbidities among psychological disorders and substance use, helping to clarify symptom centrality, highlight complex interconnections, and guide targeted interventions [11,12]. There remains an ongoing debate about whether PSU shares core mechanisms with SUD or represents distinct yet related behaviors driven by similar emotional vulnerabilities. This highlights the importance of employing network analysis methods to understand these interactions better [4,5].

While PSU is not formally recognized as a disorder in diagnostic manuals like the *Diagnostic and Statistical Manual of Mental Disorders*, *5th Edition* (the DSM-5) or the *International Classification of Diseases 11th Revision*, it exhibits similar clinical characteristics to SUD. Screening tools for PSU have been designed based on the framework of SUD, highlighting potential overlaps in the underlying mechanisms. For example, studies indicate that higher exposure to digital media is significantly correlated with the consumption of alcohol [9,13], tobacco [14], and cannabis [10].

These findings highlight potential common neurobiological and psychological mechanisms that drive both behaviors. One possible explanation is cross-sensitization, where the brain’s response to one stimulus, like substance use, increases sensitivity to other stimuli, such as pornography, gambling, PSU, social media, or online gaming [15,16,17]. This explains why people who consume alcohol or other drugs are more likely to engage in excessive use of smartphones or digital platforms, as they seek to amplify already activated reward pathways. However, not all psychoactive substances affect PSU in the same way, and the reverse is also true [8]. Central nervous system depressants, like alcohol and benzodiazepines, tend to promote relaxation and a sense of disinhibition, often leading to passive smartphone activities like watching videos or scrolling through social media as a form of escapism or immediate gratification [18]. On the other hand, stimulants such as cocaine or amphetamines increase psychomotor arousal, making it more likely for individuals to participate in dynamic and interactive activities, like social events or parties, rather than engaging in passive smartphone use [19].

While cross-sensitization partially explains the connection between PSU and SUD, a broader framework is necessary to understand how these behaviors co-occur fully. Problem-behavior theory (PBT) provides a relevant lens for exploring this relationship [20]. According to PBT, problematic behaviors—like excessive substance use, risky sexual activity, violence, and, more recently, problematic digital media use—are not isolated incidents. They represent interconnected issues rooted in common psychosocial factors, such as difficulties with self-regulation, low frustration tolerance, and increased emotional vulnerability [21]. From this standpoint, PSU and SUD are distinct but related expressions of underlying psychosocial vulnerabilities, which should be the main focus for preventive and therapeutic interventions [9].

Despite the growing interest in the link between SUD and PSU, studies have focused on general associations without exploring the specific factors that might influence this connection. For instance, Rucker et al. [10] identified a direct association between tobacco use and problematic Internet use, while connections with alcohol, marijuana, and other illicit drugs were only detected when mediated by tobacco use. However, participants were asked whether they had used certain substances in the past 30 days, using yes/no responses, limiting the study’s findings. Similarly, a large-scale study involving Japanese adolescents [9] showed a strong correlation between problematic Internet use and alcohol consumption. However, this study was limited to alcohol use, ignoring other substances. In another study with Spanish university students, alcohol use was significantly correlated with increased Internet use [22]. However, alcohol consumption was measured only by using the CAGE questionnaire, a brief four-item tool primarily meant to assess the risk of alcohol dependence.

Additionally, some authors have found that emotional states play a critical role in SUD and PSU. Individuals with higher emotional vulnerability—characterized by increased stress, anxiety, or depression—are more likely to face both SUD and PSU [19,23]. These behaviors are often seen as maladaptive coping strategies for dealing with negative emotions. Though they may provide temporary relief, PSU and SUD can perpetuate cycles of emotional dysregulation, worsening the negative symptoms associated with both disorders [24]. In this sense, network analysis presents a promising statistical approach to examine how SUD and PSU interact with emotional states through direct and indirect behavioral correlations. By looking at substance types and usage patterns—such as frequency and intensity—this method can enhance the understanding of these complex interactions, which remain an underexplored area in the literature. While previous studies have investigated the connections between substance use and behavioral addictions, there has been limited exploration of this relationship’s dynamic structure using network analysis. By identifying key symptoms and behaviors among a large group of college students, our study provides a fresh system-level perspective on how these risk factors interact in real-life situations.

Thus, the present study aimed to investigate the relationship between PSU and SUD among Brazilian university students, considering individual factors (such as emotional problems) and contextual factors (like substance use patterns). The central hypothesis was that individuals identified as having PSU would report higher levels of alcohol and drug use, as well as a greater prevalence of emotional difficulties.

## 2. Materials and Methods

### 2.1. Study Type and Participants

This study was exploratory and cross-sectional, utilizing non-probability convenience sampling. A total of 3130 university students from across Brazil (Mage = 23.6; SD = 5.34) completed an online survey that was available for two months. The sample was predominantly female (73.1%, *n* = 2287), with 26.9% identifying as male (*n* = 843). Students enrolled in technical or vocational training programs were excluded from this study. These courses typically have shorter durations and vary significantly in their curricular structures, which can differ substantially from traditional undergraduate experiences in terms of academic stress, social interactions, and daily routines. This criterion was established to maintain the homogeneity of the sample and to focus specifically on students who were regularly engaged in university-level academic environments. Additionally, we excluded those who did not fully complete the survey or whose data fell outside two standard deviations from the mean as determined by normality tests.

### 2.2. Measures

Sociodemographic: A sociodemographic questionnaire collected data on the region of residence, age, gender, sexual orientation, race/ethnicity, religious affiliation, marital status, current living arrangements, employment status, and type of higher education institution.

Alcohol Use Disorders Identification Test (AUDIT): The AUDIT is a 10-item self-report tool that assesses alcohol consumption, alcohol-related behaviors, and alcohol-related problems, using a Likert scale from 0 to 4. It includes three domains: alcohol use, dependence symptoms, and harmful consequences. The Brazilian version has demonstrated excellent internal consistency (α = 0.93; [25]). In this study, we observed the following internal consistency: α = 0.81; ω = 0.84.

Cut Down, Annoyed, Guilty, Eye-opener (CAGE): This instrument consists of four dichotomous questions (yes/no responses) used to evaluate alcohol dependency. Two positive responses are sufficient to suggest the need for further clinical investigation into alcohol dependence. This method is widely appreciated for its simplicity and effectiveness, allowing for the rapid identification of potential issues related to alcohol consumption [26].

DSM-5 Criteria for Alcohol Use Disorder: Alcohol use disorder was assessed using the DSM-5 criteria by using dichotomous questions (yes/no), which include significant updates from the DSM-IV-TR, such as the addition of craving and the removal of legal problems. Participants were classified based on the number of criteria endorsed: no problems (≤1 symptom) and mild (2–3 symptoms), moderate (4–5), or severe (6 or more) alcohol use disorder. In this study, we observed the following internal consistency: α= 0.82; ω = 0.83.

Timeline Followback Drinking (TLFB): The TLFB is a structured retrospective measure of alcohol consumption [27]. Participants reported the average number of drinks consumed each day of the last week (past 7 days). One standard drink was defined as a 355 mL can of Pilsner beer, containing approximately 8 g of pure alcohol. Consumption patterns were categorized as occurring on typical (weekends/holidays) or atypical (weekdays) drinking days.

Alcohol use patterns were further classified according to three common criteria [28,29]: (i) the WHO guidelines—weekly consumption exceeding 21 g of pure alcohol for men and 14 g for women; (ii) UK guidelines—weekly intake exceeding 14 alcohol units, regardless of gender; and (iii) a binge drinking criterion—consuming 100 g or more of alcohol on a single occasion at least once in the past three months.

Substance Use: We designed a questionnaire to assess the recreational use of various substances. Participants reported their lifetime and past-month use of alcohol, tobacco, e-cigarettes, hookah, cannabis, cocaine, amphetamines, and benzodiazepines. Items were based on the Alcohol, Smoking and Substance Involvement Screening Test (ASSIST) and the Drug Use Screening Inventory (DUSI), both of which are validated for use in Brazil [30]. This customized questionnaire was not separately validated in Brazil but was used to refine and detail our findings on substance use behaviors.

Smartphone Addiction Scale—Short Version (SAS-SV): The SAS-SV is a 10-item instrument rated on a 7-point Likert scale (0 to 6) designed to assess problematic smartphone use (PSU). It captures six behavioral domains: tolerance, loss of control, preoccupation with gratification, neglect of social relationships, functional impairments, and withdrawal symptoms. Total scores range from 0 to 60. In this study, participants scoring 33 or higher were classified into the PSU group, while those scoring 32 or lower were classified into the non-PSU group. The Brazilian version of the scale has demonstrated good reliability (α = 0.81; [31]). In this study, we observed the following internal consistency: α = 0.89; ω = 0.89.

Depression Anxiety Stress Scale (DASS-21): The DASS-21 assesses symptoms of depression, anxiety, and stress across three subscales, each with seven items rated on a 0–4 Likert scale. The Brazilian version of the DASS-21 [32] has shown strong internal consistency—depression (α = 0.90), anxiety (α = 0.83), and stress (α = 0.86). In this study, we observed the following internal consistency for depression (α = 0.92; ω = 0.93); anxiety (α = 0.85; ω = 0.85); and stress (α = 0.91; ω = 0.91).

### 2.3. Procedures

We collected data by using the SurveyMonkey^®^ platform, in which a link was created and distributed via social media and shared with class representatives through messaging apps. These representatives were encouraged to explain this study and forward the invitation to their classmates. Additionally, undergraduate program coordinators were invited to email the survey to students, emphasizing the study’s importance. The questionnaire remained available for two months, after which access was automatically closed by the system.

### 2.4. Data Analysis

We used a chi-square test to analyze categorical variables and Cramer’s V test to calculate effect size. For continuous variables, Levene’s test was performed to assess homogeneity, and the Kruskal–Wallis test was employed to assess normality. One-way ANOVA with Welch correction was applied when appropriate.

Network analysis (NA) was conducted using a Gaussian graphical model estimated via the Graphical Lasso (GLASSO) method. This approach aimed to identify both direct and indirect associations between total SAS-SV scores and substance use measures. One of the strengths of network analysis is its ability to map the interconnections between outcome variables, providing a detailed view of the system structure—in this case, including both behavioral and chemical dependencies. It also detects correlation patterns across subgroups and reveals how each variable influences the network, which traditional methods may not capture. Four centrality metrics were used to interpret the network: (i) betweenness—how often a variable serves as a bridge between other variables; (ii) closeness—how close a variable is to all others in the network; (iii) strength—the sum of the connections a variable has with others; and (iv) expected influence—the estimated impact on the network when a variable is removed.

## 3. Results

Among the total sample, 78.9% of participants (*n* = 2469) were enrolled in private universities, while 21.1% (n = 661) attended public institutions. In the current sample, we found that 46.9% of students were identified as problematic smartphone users (PSU), while the remaining 53.1% were classified as non-problematic users (nPSU). Interestingly, there was no significant difference in mean age between the two groups. However, our analysis, as illustrated in Table 1, revealed that PSU individuals were disproportionately represented among women, those without religious affiliations, students living away from their parents, and those who were unemployed or had never held a job. While these differences were statistically significant, the effect sizes—Cramer’s V ranging from 0.05 to 0.06—suggest that the associations, although small, were meaningful.

Moving on to substance use patterns over the past month, as shown in Table 2, PSU participants exhibited higher rates of consumption for various substances, particularly alcohol and illicit drugs. Notably, 56.6% of PSU students reported alcohol use, compared to 46.4% in the nPSU group, which indicates a moderate effect size (Cramer’s V = 0.10). Similar trends were observed for marijuana, amphetamines, and benzodiazepines, where prevalence rates in the PSU group were again higher, with small-to-moderate effect sizes (Cramer’s V ranging between 0.05 and 0.07). Also, cocaine use did not show significant differences between the two groups.

When examining past-year substance use (see Table 3), the disparities became even more pronounced. Alcohol use surged to 75.5% among PSU participants, in stark contrast to 63.1% among nPSU individuals, marking the highest effect size observed (Cramer’s V = 0.13). We also observed stronger associations for marijuana and e-cigarette use over this longer timeframe, with effect sizes of 0.10 and 0.09, respectively.

Table 4 explores patterns of alcohol consumption and intensity. While we did not find significant group differences based on the WHO, UK, or Russian consumption risk criteria, it was evident that PSU individuals were significantly more likely to meet the DSM-5 criteria for moderate or severe alcohol use disorder (χ^2^ = 37.9, *p* < 0.001). One-way ANOVA results showed that PSU participants scored significantly higher across all domains of the AUDIT and the total symptom count of the DSM-5. Although effect sizes were not reported directly, the data reflect consistent and substantial differences that carry practical implications for intervention strategies in this area.

**Figure 1:**
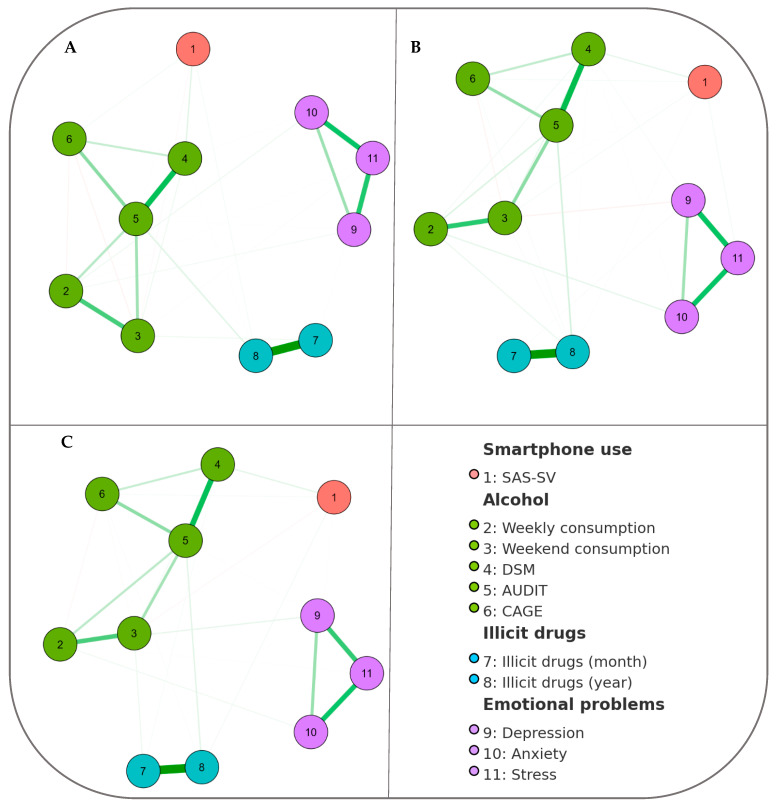
Gaussian graphical model considering 13 variables (nodes) evaluating smartphone dependence (SAS), alcohol use patterns (total weekly consumption and on typical days, total number of DSM-V symptoms for AUD, total AUDIT score, CAGE); total illicit substances consumed in the last month, and emotional problems (DASS-21 subscales). Green edges indicate positive correlations, with thicker edges representing stronger correlation intensities. (**A**) The total sample, (**B**) only men, and (**C**) only women.

Our network analysis revealed strong correlations between various nodes, particularly among factors associated with alcohol consumption patterns and the assessment tools used to evaluate these patterns, such as the AUDIT, CAGE, and DSM-5, across the total sample (Figure 1A). Notably, robust connections were also identified between recent use of illicit substances—both in the past month and the past year. Additionally, we observed significant correlations between stress and both anxiety and depression. In contrast, smartphone use (PSU) exhibited weaker correlations with other nodes within the network, a trend consistent across both male (Figure 1B) and female participants (Figure 1C).

**Figure 2:**
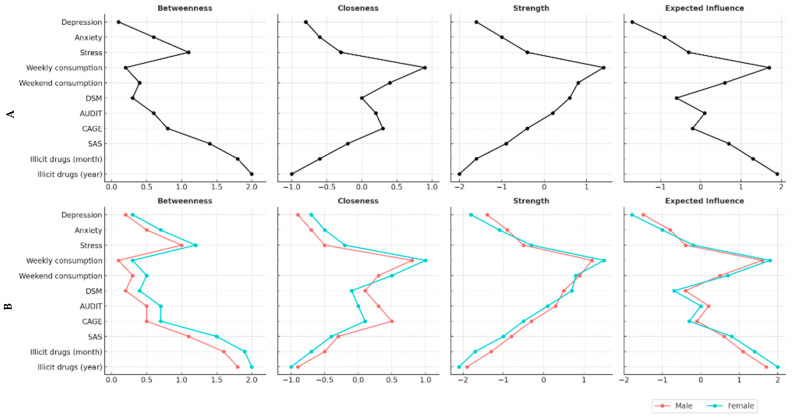
Indices of four levels of centrality considering the total sample (**A**) and participants by gender (**B**).

Figure 2 illustrates the centrality indices for all participants (2A) and further breaks these down by gender (2B). Within the broader network, alcohol consumption, measured by the AUDIT, and stress emerged as the most central constructs according to various centrality metrics, especially in terms of strength and expected influence. Furthermore, anxiety and depression demonstrated significant betweenness and closeness centralities, indicating that these variables play essential roles as connectors within the network. In contrast, smartphone addiction (SAS) and cannabis use exhibited lower centrality levels, positioning them more on the periphery of the network structure.

The gender-specific analyses revealed similar patterns across both networks; however, stress displayed slightly greater centrality among females, while the AUDIT consistently maintained its significance in both male and female groups. These findings are crucial as they reinforce our hypothesis that alcohol use and emotional distress serve as foundational elements linking problematic smartphone use (PSU) with various substance use behaviors.

## 4. Discussion

In this study, we aimed to investigate the potential connection between PSU and SUD by examining various types of substances and patterns of usage. Consistent with our hypothesis, we detected that PSU was linked to substance use behaviors and emotional distress. Specifically, we found that variables such as alcohol consumption and stress symptoms were particularly significant within this network.

Our findings indicated that around 47% of university students were classified into the PSU category, with a notably higher prevalence among women, individuals without religious ties, those living alone, and those who were unemployed or had never worked. We also found a strong correlation between SUD and PSU, which is evident in monthly and yearly consumption patterns. In terms of alcohol, statistical significance was only noted for the DSM-5 addiction criteria, AUDIT scores, and CAGE results.

In this study, we found that stress is a crucial factor coupling PSU and SUD. This suggests that interventions aimed at reducing stress could effectively address both issues. Furthermore, PSU may serve as a coping mechanism for dealing with stress related to substance use. This hypothesis demands further exploration in longitudinal studies.

Another relevant data point was that the association between PSU and substance use was stronger when annual consumption patterns were considered (Table 3), compared to monthly patterns (Table 2). This may be attributed to the fact that annual measures better capture stable, long-term behaviors, whereas monthly use might reflect episodic or situational events that participants might overlook or underestimate [1]. As such, individuals tend to have a clearer understanding of their behaviors when evaluated over a longer period, allowing them to recognize these associations more easily [33]. This finding aligns with PBT [20], which suggests that problematic behaviors often indicate deeper emotional vulnerabilities, making longer-term assessments more effective for identifying consistent behavioral patterns [18].

In the context of PBT, it is relevant to examine the roles of the three most studied substances: alcohol, tobacco, and cannabis. Our research particularly highlights the significance of alcohol, which demonstrated a notably strong link to PSU in our findings, corroborating results from other studies [5,19,24,34,35]. Specifically, we found a robust association between PSU and scores on the AUDIT, DSM-5, and CAGE assessments. These findings are important because they suggest that the relationship between PSU and alcohol consumption is not just about how often or how much someone drinks but also about the issues that arise from that consumption, as indicated by these measures [10].

This understanding fits well with the conceptual framework of various tools used to evaluate behavioral addictions, including the SAS-SV used in our study. Similarly to the AUDIT and DSM-5, the SAS-SV focuses on aspects like loss of control, excessive preoccupation, neglecting daily responsibilities, and psychosocial problems associated with PSU. The conceptual overlaps among these scales support the idea that PSU and SUD might reflect distinct yet closely related behavioral expressions stemming from the same psychosocial vulnerabilities, in line with the PBT framework [9,20,24].

When we looked at different consumption patterns, such as the international risk guidelines from the WHO and UK recommendations, including consuming ≥100 g of pure alcohol over the past three months, we found no significant associations between these risk classifications and PSU. To our knowledge, this is the first study to explore the connection between PSU and alcohol using these specific criteria. It is possible that other factors, like gender, could play a role in this relationship. For example, in the longitudinal study by Sun et al. [36], problematic Internet use was found to predict subsequent increases in substance use only among women. The data were collected from young Americans and Chinese participants, and the authors pointed out important gender differences, noting that women often have distinct patterns of Internet use (e.g., social media) and different alcohol consumption trends compared to men. They also discussed how cultural differences between American and Chinese youth might influence these associations.

Similarly, the frequency of drinking days and the amount consumed were not linked to PSU, in contrast to findings from other studies [9,10,36]. However, the network analysis indicated that weekly alcohol consumption, as assessed using the TLFB technique, had the highest centrality levels. This is significant because it implies that regular drinking patterns might have a broader impact on the various variables we studied.

One of the few studies examining PSU and SUD through network analysis, conducted by Andrade et al. [8], found that alcohol consumption also displayed high centrality levels. Interestingly, the authors noticed that alcohol was not directly linked to SUD but rather to tobacco use. Similar results were seen by Rücker et al. [10], where the authors applied a different statistical model; they found that tobacco and alcohol use had direct and indirect associations with problematic Internet use. They argued that other unmeasured variables, such as socioemotional factors, might influence the asymmetrical relationships between SUD and Internet use.

In our study, stress showed high centrality indices, especially regarding betweenness. This suggests that stress plays a critical role in linking and mediating the relationships between PSU, SUD, and other emotional variables like anxiety and depression. This is particularly relevant considering the PBT model, which posits that emotional vulnerabilities, such as poor stress management and low frustration tolerance, can help explain the coexistence of various problematic behaviors.

In a study involving over 5000 Chinese adolescents, those with higher levels of problematic Internet use reported significantly more depressive symptoms, which in turn were directly linked to increased tobacco use [14]. The authors argued that depressive symptoms might act as a connecting pathway that strengthens the relationship between problematic Internet use and smoking, underscoring the critical influence of emotional difficulties in the concurrent expression of these two problematic behaviors. In addition to validating associations earlier reported in the literature, our study makes an original contribution by mapping the relative importance of emotional and behavioral variables. This approach identifies potential intervention targets that may not be apparent in traditional linear models.

Our study has some limitations. First, being an exploratory study with a cross-sectional design prevents us from making causal inferences about the relationships identified between PSU, SUD, and emotional states. This limitation is particularly relevant in the context of network analysis, as it prevents interpretation of the directionality of associations among nodes. Second, our sample was convenience-based and comprised only Brazilian university students, limiting our findings’ applicability to other age groups, educational levels, or cultural contexts. Third, we relied entirely on self-report measures, which might introduce bias regarding how participants perceive their behaviors, particularly regarding PSU and substance use patterns. Additionally, although the adapted questionnaire for assessing substance use was based on internationally validated tools (ASSIST and DUSI), it was not formally validated in the present format for the Brazilian population. This may affect the precision of substance use estimates and limit comparability with studies using fully validated versions.

Also, the assessment of PSU was conducted solely with the SSAS-SV instrument, which may restrict our understanding of relationships between SUD and the problematic use of other digital media or specific usage contexts. Lastly, emotional states like anxiety, depression, and stress were evaluated only using DASS-21, which limits our diagnostic depth and restricts our ability to draw more detailed clinical conclusions about symptom severity or formal diagnoses.

For future research, we recommend incorporating a variety of assessment methodologies, including longitudinal designs, structured clinical interviews, and objective behavioral monitoring techniques, to strengthen methodological rigor and broaden the interpretative scope of the findings. It would also be worthwhile to study these usage patterns in adolescents or the general adult population, as digital media use and substance consumption patterns differ considerably across age groups.

## Figures and Tables

**Table 1:** Sociodemographic characteristics of participants considering those classified as problematic smartphone users (PSU, n = 1467) and non-problematic smartphone users (nPSU, n = 1663).

	PSU		nPSU				
	N	%	N	%	χ^2^	*p*	Effect
Gender					8.03	***	0.05
Male	360	24.5	483	29.0			
Female	1107	75.5	1180	71.0			
Ethnic origin					1.57	0.45	0.02
Caucasian	954	68.3	1059	66.6			
Brown	342	24.5	421	26.5			
Black	101	7.2	110	6.9			
Marital Status					2.55	0.11	0.03
Single	1191	81.2	1312	78.9			
Married	276	18.8	351	21.1			
Have a religion?					12.23	***	0.06
Yes	890	60.7	1109	66.7			
No	577	39.3	554	33.3			
Who you live with					10.6	*	0.06
Family members	869	59.2	946	56.9			
Parents	383	26.1	505	30.4			
Roommates	90	6.1	72	4.3			
Alone	125	8.5	140	8.4			
Employment status					10.01	**	0.06
Employed	544	37.1	709	42.6			
Unemployed	410	27.9	426	25.6			
Never employed	513	35.0	528	31.7			
University type					1.55	0.21	0.02
Private	1143	77.9	1326	79.7			
Public	324	22.1	337	20.3			

Notes: N = number of participants; % = frequency of participants considering the columns (research outcome); χ^2^ = chi-square test; * *p* ≤ 0.05; ** *p* ≤ 0.01; *** *p* ≤ 0.001; effect: Cramer’s V test.

**Table 2:** Substance use behavior in the last month considering participants classified as problematic smartphone users (PSU, *n* = 1467) and non-problematic smartphone users (nPSU, *n* = 1663).

	PSU		PSU				
	N	%	N	%	χ^2^	*p*	Effect
Illicit drugs					15.61	***	0.07
Yes	205	14.0	157	9.4			
No	1262	86.0	1506	90.6			
Alcohol					32.6	***	0.10
Yes	831	56.6	772	46.4			
No	636	43.4	891	53.6			
Cigarettes					3.13	0.07	0.03
Yes	184	12.5	175	10.5			
No	1283	87.5	1488	89.5			
E-cigarettes					7.44	**	0.05
Yes	99	6.7	75	4.5			
No	1368	93.3	1588	95.5			
Hookah					4.08	*	0.03
Yes	98	6.7	83	5.0			
No	1369	93.3	1580	95.0			
Marijuana					13.97	***	0.07
Yes	198	13.5	154	9.3			
No	1269	86.5	1508	90.7			
Cocaine					0.10	0.74	0.00
Yes	10	0.7	13	0.8			
No	1457	99.3	1649	99.2			
Amphetamines					9.03	**	0.05
Yes	27	1.8	11	0.7			
No	1440	98.2	1652	99.3			
Benzodiazepines					7.91	**	0.05
Yes	58	4.0	37	2.2			
No	1409	96.0	1626	97.8			

Notes: legend: N = number of participants; % = frequency of participants considering the columns (research outcome); χ^2^ = chi-square test; * *p* ≤ 0.05; ** *p* ≤ 0.01; *** *p* ≤ 0.001; effect: Cramer’s V test.

**Table 3:** Substance use behavior in the last year considering participants classified as problematic smartphone users (PSU, *n* = 1467) and non-problematic smartphone users (nPSU, *n* = 1663).

	UPS		nUPS				
	N	%	N	%	χ^2^	*p*	Effect
Illicit drugs					31.7	***	0.10
Yes	375	25.6	288	17.3			
No	1092	74.4	1375	82.4			
Alcohol					55.7	***	0.13
Yes	1107	75.5	1049	63.1			
No	360	24.5	614	36.9			
Cigarettes					6.93	**	0.05
Yes	316	21.5	296	17.8			
No	1151	78.5	1367	82.2			
E-cigarettes					23.2	***	0.09
Yes	222	15.1	158	9.5			
No	1245	84.9	1505	90.5			
Hookah					20.4	***	0.08
Yes	245	16.7	185	11.1			
No	1222	83.3	1478	88.9			
Marijuana					29.3	***	0.10
Yes	367	25.0	285	17.1			
No	1100	75.0	1378	82.9			
Cocaine					1.65	0.20	0.02
Yes	31	2.1	25	1.5			
No	1436	97.9	1638	98.5			
Amphetamines					17.5	***	0.07
Yes	60	4.1	27	1.6			
No	1407	95.9	1636	98.4			
Benzodiazepines					16.6	***	0.07
Yes	113	7.7	71	4.3			
No	1354	92.3	1592	95.7			

Notes: *N =* number of participants; *% =* frequency of participants considering the columns (research outcome); *χ*^2^ *=* chi-square test; ** *p ≤* 0.01; *** *p ≤* 0.001; effect: Cramer’s V test.

**Table 4:** Different criteria regarding alcohol consumption and consumption intensity considering participants classified as problematic smartphone users (PSU, *n =* 1467) and non-problematic smartphone users (nPSU, *n =* 1663).

	UPS		nUPS			
	N	%	N	%	χ^2^	*p*
Consumption Criteria						
WHO					2.39	0.12
At risk	226	19.6	188	17.1		
No risk	926	80.4	912	82.9		
UK					1.73	0.21
At risk	250	21.7	214	19.5		
No risk	902	78.3	886	80.5		
100 g or more of alcohol					2.58	0.10
0 g	774	66.9	767	70.0		
≥100 g	383	33.1	328	30.0		
DSM-5					37.9	***
No addiction	683	59.0	780	71.2		
Mild addiction	246	21.3	175	16.0		
Moderate addiction	117	10.1	75	6.8		
Severe addiction	111	9.6	65	5.9		
	M	SD	M	SD	F	*p*
AUDIT						
Total score	6.31	5.52	5.20	4.70	26.3	***
Alcohol consumption	3.79	2.42	3.53	2.34	7.14	**
Drinking behavior	0.55	1.37	0.31	1.05	20.8	***
Alcohol-related problems	1.96	2.83	1.36	2.33	30.45	***
DSM-5 (Total symptoms)	1.93	2.40	1.35	2.04	38.23	***
CAGE						
Total score	0.65	0.87	0.50	0.76	16.67	***
Weekend alcohol use	3.31	4.34	2.88	4.13	5.73	*

Notes: *N =* number of participants; *% =* frequency of participants considering the columns (research outcome); *χ*^2^ *=* chi-square test; *M =* mean; *SD =* standard deviation; *F =* one-way analysis of variance (ANOVA); * p ≤ 0.05; ** *p ≤* 0.01; *** *p ≤* 0.001.

## Data Availability

The data that support the findings of this study can be requested from the authors.
